# White Matter Tracts Associated With Deep Brain Stimulation Targets in Major Depressive Disorder: A Systematic Review

**DOI:** 10.3389/fpsyt.2022.806916

**Published:** 2022-04-28

**Authors:** Qun Yu, Xinxia Guo, Zhoule Zhu, Chen Feng, Hongjie Jiang, Zhe Zheng, Jianmin Zhang, Junming Zhu, Hemmings Wu

**Affiliations:** Department of Neurosurgery, The Second Affiliated Hospital, Zhejiang University School of Medicine, Hangzhou, China

**Keywords:** deep brain stimulation, diffusion tensor imaging, major depressive disorder, white matter tracks, review

## Abstract

**Background:**

Deep brain stimulation (DBS) has been proposed as a last-resort treatment for major depressive disorder (MDD) and has shown potential antidepressant effects in multiple clinical trials. However, the clinical effects of DBS for MDD are inconsistent and suboptimal, with 30–70% responder rates. The currently used DBS targets for MDD are not individualized, which may account for suboptimal effect.

**Objective:**

We aim to review and summarize currently used DBS targets for MDD and relevant diffusion tensor imaging (DTI) studies.

**Methods:**

A literature search of the currently used DBS targets for MDD, including clinical trials, case reports and anatomy, was performed. We also performed a literature search on DTI studies in MDD.

**Results:**

A total of 95 studies are eligible for our review, including 51 DBS studies, and 44 DTI studies. There are 7 brain structures targeted for MDD DBS, and 9 white matter tracts with microstructural abnormalities reported in MDD. These DBS targets modulate different brain regions implicated in distinguished dysfunctional brain circuits, consistent with DTI findings in MDD.

**Conclusions:**

In this review, we propose a taxonomy of DBS targets for MDD. These results imply that clinical characteristics and white matter tracts abnormalities may serve as valuable supplements in future personalized DBS for MDD.

## Introduction

Major depressive disorder (MDD) is a type of mood disorder characterized by significant and persistent depressed mood with various degrees of cognitive and behavioral changes. According to the World Health Organization, MDD will be ranked first in disease burden worldwide by 2030 (1). The effectiveness of antidepressant medications is limited, and up to 35% of MDD cases remain recurrent and resistant to medications (2). Deep brain stimulation (DBS), as a promising neuromodulation therapy, has shown potential antidepressant effects in otherwise-refractory MDD.

DBS exerts electric impulse to modulate neuronal activity and dysfunctional brain circuits, and serves as a therapy for various neurological disorders, including movement disorders, epilepsy, pain, and psychiatric disorders. DBS was first tested as a potential treatment for MDD in a clinical setting in 2005 (3). Since then, open-label clinical trials are performed *via* different targets with response rates varying from 36 to 60% at 1-year follow-up (4). Recently, two double-blind, randomized, sham-controlled trials failed to show statistically significant improvement in terms of antidepressant efficacy of DBS for MDD in stimulation group vs. sham group (5, 6). As a result, DBS remains an investigational treatment for MDD. There are many contributing factors, one of which is the diversity of brain circuits modulated by DBS between individuals from a group of heterogeneous MDD patients. Recent studies indicate that white matter tracts (WMT) play a crucial role in DBS for MDD (7), and DBS alleviates depressive symptoms by modulating neural network involved *via* fiber connections (8). However, currently used DBS targets for MDD are not based on personalized circuit targeting, which may lead to inaccurate modulation and suboptimal effects (9).

Diffusion weight imaging (DWI) is a non-invasive technique sensitive to water movement to quantify the tissue diffusion rate within imaging voxel. DTI is a specific type of modeling of the DWI using at least six diffusion measurements and directions. It can be used for studying WMT microintegrity by properties, such as fractional anisotropy (FA), mean diffusivity (MD), apparent diffusion coefficient (ADC), and axial and radial diffusivity in disease and healthy control. Regions of interest (ROIs), voxel-based analysis and tract-based spatial statistics (TBSS) are typically performed for group statistical analysis after obtaining parametric maps (10). Overall, DTI is a promising tool for studying *in vivo* WMT microstructure abnormality and neural circuit dysfunction, which are implicated in MDD. This conceptualize new insights into different models of dysfunctional brain circuits in MDD patients, including default mode, salience, negative affect, positive affect, attention, and cognitive control, while suited treatment is proposed for each model (11). DTI can also be used for tractography based on the primary eigenvector of diffusion to obtain three-dimensional representations of WMT. Tractography based on DTI unravels the relationship between DBS targets and associated WMT, which may elucidate the action mechanism of DBS for each target, and provide a practical utility for personalized targeting. There are two kinds of tractography methods: probabilistic and deterministic. Deterministic tractography reconstruct one fiber from each seed based on maximum vector, while probabilistic approaches take into account the uncertainty of the estimation and provides probability maps for each seed. Connectomic DBS is gradually receiving more and more endorsement for its ability to achieve network-level targeting. Here, we speculate that different DBS targets for MDD modulate different dysfunctional neural circuits, and conduct a systematic review of existing DBS targets for MDD to present a taxonomy of modulated WMT and dysfunctional brain circuits.

## Methods

This systematic review was conducted following the 2020 PRISMA guideline (12). To summarize the DBS clinical trials for MDD, articles and review papers were searched from January 2005 to June 2021 using PubMed. Combinations of 3 medical subject heading terms (i.e., “depressive disorder,” “major, treatment-resistant,” and “depression”) and 3 keywords, including “deep brain stimulation,” “DBS,” and “electrical stimulation”, were used as search criteria. The reference lists of relevant articles were also screened. The articles include case reports, open-label trials and randomized controlled trials, using DBS treatment for MDD. A total of 51 clinical studies of DBS for MDD were matched. Then, the latter set of keywords was replaced by the terms “diffusion tensor imaging,” “DTI,” or “white matter tracts” in the search process for DTI-relevant literature from January 2010 to June 2021. The articles using DTI to study abnormal WMT in MDD were included, and a total of 44 MDD DTI studies were matched. The search was limited to articles published in English. Three co-authors (QY, XXG, and ZLZ) searched and assessed studies independently to ensure accuracy and completeness, and to reduce selection bias. Different terminologies are used in different DTI studies to describe similar WMT (e.g., genu of the cingulate cortex vs. forceps minor), which remains a source of information bias despite the co-authors' best effort to eliminate it.

## Results

### Review of the DBS Targets and Associated WMT

DBS is used as a treatment for MDD since 2005 (3). Here we summarize currently used DBS targets for MDD as well as its associated WMT through anatomic and connectomic studies. An overview of DBS targets for MDD is listed in Supplementary Table 1.

### Subcallosal Cingulate Gyrus (SCG)

In 2005, Mayberg et al. first proposed the SCG as an effective target for treating MDD, with four of six patients experiencing remission at 6 months (3). A series of open-label trials followed, with moderately satisfactory effects, showing expected responder rates between 55 and 75% (13–15). Several case reports also demonstrated the effectiveness of SCG-DBS (16–21). In a preliminary, double-blind, randomized, sham-controlled, crossover trial, 4 out of 5 patients were remitted after 6 months of stimulation, and none of them experienced relapse (22). The therapeutic efficacy varies with the length of stimulation time. An immediate antidepressive consequence of SCG-DBS was reported in an open-label trial (23). In the studies by Kennedy et al. and Holtzheimer et al., considerable responsiveness and remission rates were observed even 3–6 years after DBS implantation (14). Moreover, an open-label study reported that 28 participants experienced a robust and sustained antidepressant response to SCG-DBS in a 2–8 years observation (24). On the other hand, Merkl et al. reported that merely 33% of participants showed an antidepressive response within 24–36 weeks, and there was no significant difference of effectiveness between the active and sham groups (25). In addition, a randomized, double-blind, sham-controlled trial failed to demonstrate significant antidepressant efficacy of SCG-DBS during double-blind period (6). Similarly, another double-blind study of 8 MDD patients showed no significant difference in responder rates in SCG-DBS vs. sham (26). Altogether, these data suggest that SCG-DBS could be efficacious for the management of MDD, but how to improve the responder rate effectively remains a challenge for clinicians.

The SCG is located at the center of a rich network of fiber connections including the cingulum bundle (CB), forceps minor (FM), and uncinate fasciculus (UF), and projects to the orbitofrontal cortex (OFC), anterior cingulate cortex (ACC), thalamus, ventral striatum, hippocampus, amygdala, and temporal lobe (27). Howell et al. suggested that CB and FM were the most likely targeted WMT for SCG-DBS (28). Studies suggested that medial frontal cortex *via* FM and UF, cingulate cortex *via* CB, and subcortical nuclei were the critical activation volumes for responders (7), and antidepressant responses were only demonstrated in the patient who had strong connectivity of the stimulation areas to the medial prefrontal cortex (mPFC) (29). Riva-Posse et al. and Choi et al. used post-operative and intraoperative tractography mapping of the “depression switch” of SCG-DBS and demonstrated that responders showed distinct activation pathways from the ventromedial prefrontal cortex (vmPFC) *via* the FM and UF and the rostral and dorsal cingulate cortex *via* the CB (7, 30). For this purpose, Riva-Posse et al. used tractography-based surgical targeting for CB, UF, and FM, and reported 9 of 11 patients responding to the treatment at 2 years (31). Lujan et al. used a tractography-activation model tool and found that the most therapeutic electrode contacts primarily projected to the brain regions associated with the vmPFC, nucleus accumbens (NAcc), and CB, and any small differences in the electrode site may produce substantial differences in the activated pathways (32).

### Medial Forebrain Bundle (MFB)

Schlaepfer et al. first reported the clinical antidepressant effect of MFB-DBS (33). Fenoy et al. assessed the efficacy of MFB-DBS and reported two out of three patients continued to have more than 80% decrease in Montgomery Asberg Depression Rating Scale (MADRS) scores at 26 weeks. Fenoy et al. further reported that four out of five patients had a 70% decrease in MADRS scores relative to baseline at 52 weeks in a longitudinal study (34). An open-label trial of DBS of the MFB in MDD suggested that 6 out of 8 participants responded at 1 year, including 4 patients who achieved total remission (35). Furthermore, the antidepressant efficacy remained stable for up to 4 years, which suggested acute and sustained antidepressant efficacy (36). Recently, Coenen et al. provided long-term data for a small phase I, randomized controlled clinical trial. They reported that all patients reached the response criterion, 63% responded within a week, and 50% were classified as remitters after 12 months of stimulation (37). No evidence of side effects has yet been reported, but a case study reported that a patient suffered from blurred vision after 10 months (38).

The MFB incorporates mesolimbic pathways that originate from the ventral tegmental area (VTA) and projects to the NAcc and the prefrontal cortex (PFC). It is a central component of the mesolimbic–mesocortical dopamine reward system (39, 40). MFB-DBS could activate the mesocorticolimbic system by increasing neuronal activity through the modulation of dopaminergic and glutamatergic neurotransmission (41). MFB serves both as a specific stimulation target and the center of the reward pathway simultaneously. Nonetheless, the complex midbrain area contains tightly intertwined myelinated fibers. There is an ongoing debate about which WMT actually contributes to anti-depressive mechanism. The VTA dopaminergic axons do not travel within internal capsule, while superolateral MFB (slMFB), a branch undercuts the thalamus, moving laterally toward the internal capsule in its ventral portion, and then goes profoundly into Nacc and PFC, may contribute to the antidepressant effect (42–44).

### Nucleus Accumbens

The effectiveness of NAcc-DBS has been reported in several trials (45, 46). An improvement in the Hamilton Depression Rating Scale (HDRS) was observed in 3 out of 4 patients in an open-label trial, whose moods improved simultaneously (45). In addition, an open-label trial with 10 patients reported 50% responder rates of HDRS scores 1 year after the implantation of NAcc-DBS (47). Long-term trial reported a sustained antidepressant effect of up to 4 years, with 5 out of 11 patients reaching the response criterion (46). Meanwhile, the neuropsychological safety of NAcc-DBS for MDD was demonstrated in a 12-month follow-up study (48).

Anatomically, the NAcc is divided into the core and the shell, which receive motor and limbic system information, respectively (49). In general, efferents of the NAcc project to the cingulate gyrus, ventral pallidum, and thalamus (50). The afferents to the NAcc are glutamatergic from the PFC, hippocampus, and amygdala, which excites neurons of the NAcc to establish roles in the neurocircuitry of pleasure and reward (51). The NAcc and SCG have close relationships with direct fiber connections, and the underlying tracts are the CB, FM, and a part of the UF (52). The strength of the connections between the NAcc stimulation sites and the medial and lateral PFC significantly predicted clinical improvement in obsessive-compulsive disorder (OCD) based on diffusion magnetic resonance imaging (53), but similar studies have not been reported in MDD yet. As the clinical effect and mechanism of NAcc-DBS remain a matter of debate, pilot studies are needed to prove effective stimulation target localization and possible brain networks in NAcc-DBS. In anatomical studies, the fiber pathway passing adjacent to or connecting to the NAcc has eight tracts, including the CB, UF, and FM, forming a capsule around the sides of the Nacc (52). Another study combining anatomical structure and tractography finds NAcc-DBS involves modulation of the anterior thalamic radiation (ATR), inferior fronto-occipital fasciculus (IFOF), and inferior longitudinal fasciculus (ILF) (54). MFB pass through the NAcc, and then the fibers extend toward the OFC and PFC (40).

### Ventral Capsule/Ventral Striatum (VS/VS)

The VC/VS is crucial in the cortico-striatal-pallidal neural circuits and is vital in reward and motivation (55). VC/VS-DBS was first employed in patients with OCD. During these studies, it was found that the subjects' comorbid depressive symptoms also significantly improved (56, 57). These results led to an initial open-label trial of VC/VS-DBS for MDD, and the trials showed favorable response rates (response rates with the HDRS were 40% at 6 months and 53.3% at last follow-up) (58, 59). Meanwhile, a case report described a single responder after VC/VS-DBS who showed cessation of smoking, which indicated that VC/VS-DBS might compensate for reward deficits and lead to reduced smoking (60). These positive results led to randomized controlled trials. However, the results were contradictory. Dougherty et al. suggested VC/VS-DBS is not an efficacious therapy for MDD, as the response rates at 12, 18, and 24 months during the continuation phase were 20%, 26.7%, and 23.3%, respectively (5). Additionally, the application of VC/VS-DBS was also referred to as ventral-ALIC-DBS (a brain structure that is slightly anterior and ventral to the VC/VS) in some studies (61–63). Bergfeld et al. showed that ventral-ALIC-DBS resulted in a significant decrease in depressive symptoms in 10 out of 25 patients and was well-tolerated (63).

Different targets may modulate the same neural network that is responsible for clinical improvement. Studies by Li et al. and van der Vlis et al. showed that a subpart of the ALIC, which connects areas of the prefrontal cortex with the subthalamic nucleus and medial nucleus of the thalamus, is associated with an effective response in VC/VS stimulation for refractory OCD (64, 65). Previous studies using DTI showed that the ventral ALIC contains two fiber bundles: the ATR and the slMFB, which are implicated in reward and punishment functions (42). slMFB emerged as a target for the treatment of MDD and provided dopaminergic input from the brainstem. Another study argued that clinical behavioral improvements with either ventral-ALIC- or NAcc-DBS result from activation of the slMFB (66). Our previous tractography study showed that ALIC-DBS also activated IFOF and FM, which projected to the prefrontal cortex, ventral striatum, and occipital lobe (54).

### Bed Nucleus of the Stria Terminalis (BNST)

To date, only few clinical reports of BNST-DBS for MDD are available. In a case report, a patient with severe MDD combined with anorexia nervosa received a DBS implant in the MFB but treatment was discontinued due to the side effects of blurred vision after 2 years. BNST-DBS was then employed, which resulted in profound and persistent improvement (38). In another case study, the authors had a longitudinal neuropsychological assessment performed for an MDD patient following 12 months of BNST-DBS, and significant clinical improvements in mood and anxiety were indicated post-stimulation (67). A pilot open-label clinical study was conducted in five patients, which presented sustained remission of depressive symptoms in two participants, substantial antidepressant effects in two patients, but had minimal therapeutic effects in one patient (68). Neumann et al. recorded local field potential activity in 7 MDD patients who received DBS electrode implants in the BNST and proposed that α-activity in the limbic system may be a biomarker of symptom severity in MDD (69). BNST is a potential target for MDD, but further exploration is warranted in larger, well-designed clinical trials.

The BNST, located in the immediate vicinity of the VC/VS and NAcc regions, is part of the limbic system (70) and has projections to many structures associated with reward, stress, and anxiety processing (71). An in-depth dissection of the structural connectivity of the BNST is of utter importance to decipher its role in depression. Avery et al. showed structural and functional connections with BNST convergence in the NAcc, thalamus, hippocampus, pallidum, caudate, and putamen *via* stria teminalis (72). Moreover, the BNST is connected to several brainstem structures *via* the MFB and the periventricular system. Kruger et al. used probabilistic fiber tracking methods to examine the connectivity mode of the human BNST *in vivo*, and there were three distinct pathways: the stria terminalis as a posterior pathway to the lateral amygdala, a ventral pathway toward the hypothalamus, and the medial amygdala *via* the ansa peduncularis (73). These findings suggested that BNST-DBS may produce modulatory effects on the cortico-subcortical and slMFB reward circuit.

### Inferior Thalamic Peduncle (ITP)

Several case reports have described the outcomes of ITP-DBS in two patients with MDD with favorable outcomes (74–76). More recently, in a double-blind crossover study, the effects of DBS at the ITP and ALIC-BNST targets were compared in patients suffering from MDD. Although both ITP and ALIC-BNST stimulation may alleviate depressive symptoms, only 1 patient out of 7 preferred ITP over ALIC-BNST stimulation (77).

The ITP is a structure of WM fibers that transmits bidirectional information from the midline and intralaminar thalamic nuclei of the non-specific thalamic system to the OFC (75). The disruption of the thalamo-orbitofrontal system ameliorates depressive symptoms, and the ITP together with the nucleus reticularis thalami plays a vital role in the pathophysiology of MDD (78). The ITP-DBS activate fibers engaged in ATR, MFB, and IFOF, which are consistent with the above anatomical structure involved in the thalamo-orbitofrontal circuit.

### Lateral Habenula (LH)

Sartorius et al. firstly reported a significant remission of depressive symptoms after 4 months of LH-DBS in a patient with MDD, and an obvious rekindling of depressive symptoms occurred after erroneous suspension of the stimulation (79). More precisely, they put electrodes into the main limbic afferent WMT of the LHb (stria medullaris) for two participants in the same trial. Both of them had improvements of more than 50% on a depressive symptom scale (80). Recently, a case report revealed that a patient with DBS of bilateral LH achieved significant clinical improvement at 12 weeks follow-up (81). They demonstrated the feasibility LH-DBS for MDD, but larger clinical trials are necessary to confirm its efficacy.

The LH is a compact nucleus that appears as a triangular ridge stretching into the third ventricle on the dorsomedial surface of the caudal thalamus (82). It receives the afferent pathway of the limbic system *via* the stria medullaris from the amygdala, and provides an efferent pathway to the BNST and dorsal raphe nucleus. Then, these fibers project to several target brain regions, such as the hippocampus, hypothalamus, amygdala, and cerebral cortex, ultimately playing an important role in emotion regulation.

The halt of a pivotal clinical trial of DBS for MDD marks the urgent need of understanding the action mechanism of DBS for MDD (6). Recent studies reveal that WMT connectivity plays an essential role in the clinical effect of DBS for MDD. Riva-Posse et al. identify four fiber pathways associated with antidepressant efficacy in SCG-DBS (7). Similar findings are reported by Lujan et al. (32). In addition, Coenen et al. propose that targeting superolateral branch of MFB is critical for effective MFB-DBS (83). These findings of association between WMT connectivity and clinical effect form a hypothesis that DBS for MDD exert antidepressant efficacy *via* WMT modulation, especially modulation of the abnormal WMT in MDD. In the following review, we summarize abnormal WMT in MDD based on DTI studies to reveal relevant WMT in DBS for MDD.

### Review of Abnormal WMT Associated With MDD

DTI is a variant of diffusion-weighted imaging, which is capable to assess microstructural changes in the brain using water molecule degree of anisotropy and structural orientation in each voxel. It uncovers different fiber connectivity integrity between diseased and healthy subjects by quantitative power. Voxel-based analysis and tract-based spatial statistics can be applied to extract summary measures from brain regions of interest (84). In this part, we summarize the abnormal WMT in MDD discovered in previous DTI studies (Supplementary Table 2).

### Cingulum Bundle

CB runs through the cingulate gyrus superior to the corpus callosum, connecting the medial frontal, parietal, occipital, and temporal lobes, and the cingulate cortex. One previous study reported increased FA in the left posterior part of the CB (85). The majority of articles reported reduced FA values in MDD patients. These fibers are in the left CB (86, 87), anterior CB (85, 88) and bilateral CB (89–91). Another two studies by Zhang et al. and Carballedo et al. found that the CB had no alteration in FA and its subregions (92, 93). In summary, most studies have shown that FA is reduced in the CB in patients with MDD.

### Medial Forebrain Bundle

MFB is an important pathway for connecting the limbic forebrain, midbrain, and cerebellum, and plays a key role in the reward circuit (42). The main tract splits into two distinct directions through the VTA (42). The inferomedial MFB traces the wall of the third ventricle anteriorly until reaching the lateral hypothalamus, and the slMFB branches into the NAcc, and then project out to the OFC and PFC (39, 42). Brache et al. reported reduced FA in the right VTA-lOFC (lateral OFC) and VTA-dlPFC (dorsal lateral prefrontal cortex) connections in melancholic patients (94). In addition, a battery of DTI studies reported reduced FA in depressed patients in the left (95, 96), right (97), and bilateral ALIC (98–100). Similarly, decreased FA values were reported in relevant frontal brain regions (100–103). These studies may reflect white matter microstructure alterations of the slMFB. However, one tractography study showed that the microstructure of the MFB in remitted depressed participants did not differ from participants without history of depression (104). Identification of MFB can be technically challenging due to extensive overlap of different fibers. For instance, the ATR is located closely and overlaps partially with the slMFB (42). Thus, further tractography studies should explore the differential role of ATR and slMFB in depression. Innovative fiber tracking techniques may provide more accurate fiber identification results in regions with crossing fibers (105).

### Uncinate Fasciculus

UF is a significant bundle that connects the anterior temporal lobe, including the hippocampus and amygdala, with the medial and lateral frontal cortex. Several studies have suggested that the FA value in the UF is reduced in patients with depression, including the left (106), right (107), and bilateral UF (87, 89, 91, 108, 109). However, increase in FA has also been reported (110). Further evidence for microstructural changes in the UF may arise from alterations in the temporal and frontal brain areas, potentially incorporating the UF. Reduced FA values in the frontal regions have been reported in the left middle frontal gyrus (101), right frontal lobe (102, 111), and bilateral frontal regions (99, 100, 103, 112). Furthermore, reduced FA in the left middle frontal gyrus and the right inferior frontal gyrus was found in unipolar depressed patients compared with healthy controls (113). Likewise, reduced FA values were found in the temporal lobe, such as the right parahippocampal gyrus (95), bilateral temporal white matter (103), left limbic lobe uncus (101) and bilateral temporal lobe (108).

### Forceps Minor

FM courses along the anteromedial surface of the NAcc toward the frontal poles to connect to the prefrontal and medial orbitofrontal brain regions, and the NAcc from side to side (52). Given that the vital function of the PFC and the NAcc are implicated in reward-processing incentives, it is not surprising that the broken integrity of the FM may lead to the development of MDD. Specifically, FA alteration in corpus callosum was found to be the most common results in a meta-analysis (86, 114). Yang et al. found that first-episode medication-naive MDD patients exhibited reduced FA in the left FM compared with healthy controls, and the mean FA values were significantly correlated with anhedonia (86). Meanwhile, Seok et al. and Murphy et al. reported that FA reductions in the bilateral FM, which connected the medial sides of the PFC, were known to be involved in depressive symptoms (88, 91). Another study was employed to demonstrate the abnormalities of the genu of the corpus callosum, which showed that reduced FA may contribute to the pathogenesis of treatment-responsive MDD (97). A similar finding was reported in a study by Lu et al., which showed diminished integrity within the genu of the corpus callosum and the FM in young healthy subjects with high-trait anxiety (115).

### Anterior Thalamic Radiation

The ATR connects the prefrontal lobe (mainly in the dlPFC) and the thalamus through the ALIC. It is difficult to identify the two fibers in the ALIC, MFB, and ATR, but they have different functional roles in the pathogenetic mechanism of MDD (42). Previous studies have found reduced FA in the left ATR, and the abnormal diffusion portion was primarily located in the dlPFC area (107). Walther et al. delineated that FA values in MDD in the left ATR are decreased (116). Bessette et al. and Lai and Wu reported that white matter integrity in patients with MDD have lower FA values in the right ATR (99, 117). Moreover, researches showed that in participants with anxiety-related disorders, the integrity of the bilateral ATR is diminished (115, 118). Owing to the difficulty in distinguishing the ATR and MFB among the ALIC section, some studies suggested that ALIC changes may also contain areas of the ATR (95).

### Inferior Fronto-Occipital Fasciculus

The IFOF, which plays a pivotal role in frontal-subcortical circuits, projects from the occipital lobe, striatum, and thalamus into the frontal cortex. A meta-analysis of DTI studies in patients with MDD showed reduced FA in the IFOF traversing the right fusiform gyrus of the temporal lobe and the ILF (119). Several DTI studies demonstrated reduced FA in the IFOF in MDD patients (8, 87, 89, 99, 120–122). Recently, Sugimoto et al. found that the FA values of the bilateral IFOF and the genu of the corpus callosum in MDD patients were significantly decreased and inversely correlated with IL-1β levels (microstructural changes in the IFOF and the genu of the corpus callosum are associated with high IL-1β levels in the early stage of MDD) (8). Compared with controls, Wang et al. found decreased FA in right IFOF in late-onset depression. The results from this study also showed that WMT structural connectomic changes correlated with cognitive deficits (123).

### Inferior Longitudinal Fasciculus

The ILF connects the occipital and anterior temporal lobes and projects to the lateral and medial anterior temporal regions. The investigation of the ILF suggested that it may be involved in facial recognition, visual perception, reading, and visual memory (124). While the function of the ILF is poorly understood, several studies found reduced FA in the ILF in MDD patients (119, 125). Liang et al. reported reduced FA in the bilateral ILF and part of the left ILF in two MDD subtypes (subgroup 1, deficits in sustained attention and delayed memory; subgroup 2, dysfunction in delayed memory) (125). Zheng et al. also reported reduced FA in the left ILF in MDD (126).

### Corticospinal Tract

The CST runs through the internal capsule, which projects into the basal ganglia and separates the thalamus from the putamen, globus pallidus, and caudate nucleus, and is associated with MFB. The CST is involved in the processing and coordination of sensorimotor information that may be associated with motor retardation and slower reaction times in MDD (106). Previous studies reported reduced FA in the CST in adolescents and children with MDD (89, 99). However, the functional significance is yet to be revealed. Vilgis et al. found reduced FA in the left CST, and a significant positive association with anxious-depressed symptoms by exploratory *post-hoc* analysis (106). Recently, Liang et al. reported widespread FA reduction in the superior portion of the bilateral CST in patients with MDD who suffered more deficits in sustained attention and delayed visual memory (125). Reduced FA in the bilateral CST was also reported in drug-naive patients with MDD (8).

### Superior Longitudinal Fasciculus

The SLF connects the PFC, temporal lobe, occipital lobe, parietal lobe and limbic system, and plays an important role in emotion regulation and cognitive function (87, 90, 107). Several DTI studies reported decreased FA in the SLF in MDD compared to controls (88, 90, 91, 111, 117, 122, 123, 127, 128). Previous studies showed that increased depression severity was negatively correlated with decreased integrity in the SLF (129, 130). Liang et al. revealed widespread disruption in the bilateral SLF specifically in the subgroup of deficit in attention and memory (125). Deng et al. reported increased FA in the left SLF of the frontal lobe in MDD patients compared to healthy controls (107).

### Each DBS Target Corresponds to Dysfunctional Brain Regions and Circuits Associated With Different MDD Subtypes

**Figure 1:**
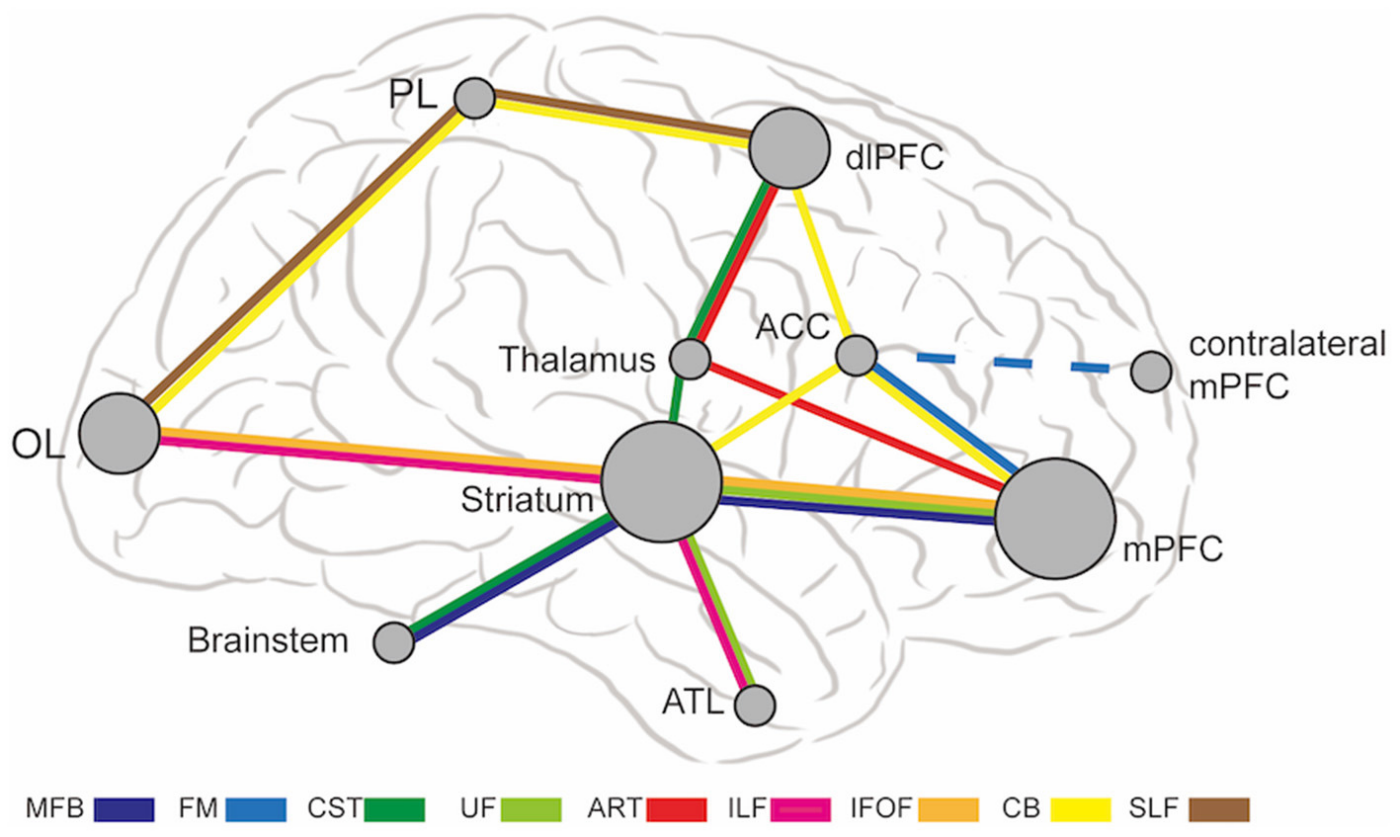
The abnormal WMT and associated brain regions in MDD. The brain region node size represents the number of passing WMT. ACC, anterior cingulate cortex; ATL, anterior temporal lobe; ATR, anterior thalamic radiation; CB, cingulum bundle; CST, corticospinal tract; dlPFC, dorsal lateral prefrontal cortex; FM, forceps minor; IFOF, inferior fronto-occipital fasciculus; ILF, inferior longitudinal fasciculus; MFB, medial forebrain bundle; mPFC, medial prefrontal cortex; OL, occipital lobes; PL, parietal lobes; SLF, superior longitudinal fasciculus; UF, uncinate fasciculus.

The abnormal WMT in MDD causes dysfunctional connections in multiple brain regions, including medial PFC, dlPFC, ACC, striatum, thalamus, anterior temporal lobe, parietal lobe, occipital lobe, and brainstem. Figure 1 shows a schematic diagram of abnormal WMT and corresponding brain regions in MDD. The mPFC and striatum are two hub dysfunctional brain regions in MDD, which correspond to SCG, VC/VS, BNST, and NAcc target locations. SCG and VC/VS are the most commonly used DBS targets for MDD in previous studies. Thalamus and brainstem are also implicated in MDD, which correspond to MFB, ITP, and LH target locations. Several brain regions, including occipital lobes, parietal lobes, dlPFC, ACC, and anterior temporal lobes are not directly targeted by DBS, but can be modulated by connected WMT.

**Figure 2:**
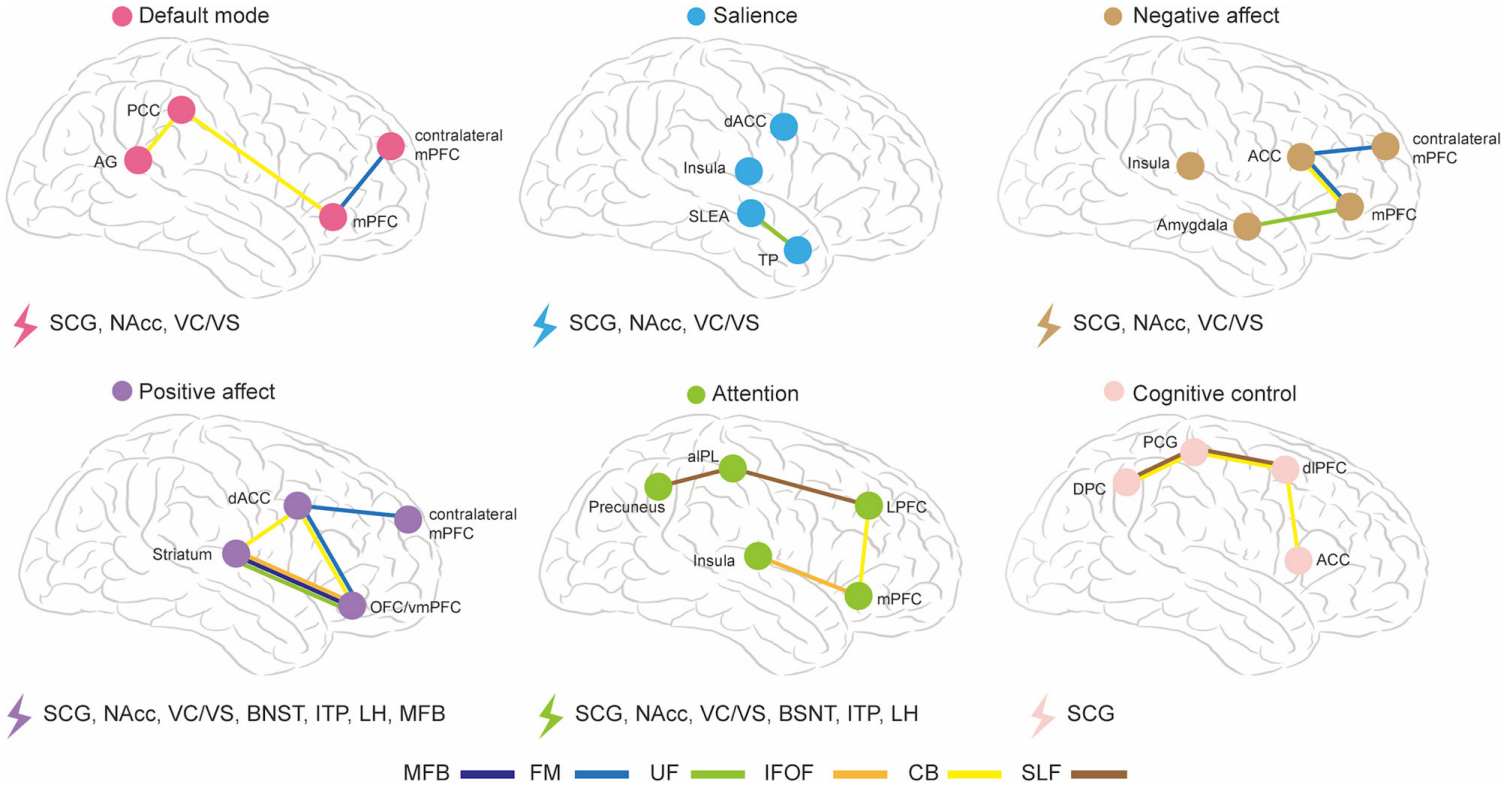
The abnormal WMT in MDD and their implications in different DBS targets and dysfunctional brain circuits. ACC, anterior cingulate cortex; AG, angular gyrus; aIPL, anterior inferior parietal lobule; BNST, bed nucleus of the stria terminalis; CB, cingulum bundle; dACC, dorsal anterior cingulate cortex; dlPFC, dorsal lateral prefrontal cortex; DPC, dorsal parietal cortex; FM, forceps minor; IFOF, inferior fronto-occipital fasciculus; ITP, inferior thalamic peduncle; LH, lateral habenula; LPFC, lateral prefrontal cortex; MFB, medial forebrain bundle; mPFC, medial prefrontal cortex; NAcc, nucleus accumbens; OFC, orbitofrontal cortex; PCC, posterior cingulate cortex; PCG, precentral gyrus; SCG, subcallosal cingulate gyrus; SLF, superior longitudinal fasciculus; TP, temporal pole; UF, uncinate fasciculus; VC/VS, ventral capsule-ventral striatum; vmPFC, ventromedial prefrontal cortex.

The abnormal WMT found in DTI studies is inconsistent, which may due to mixed symptomatology and biotypes, which are likely to overlap in individuals. Different dysfunctional brain circuits underlie different biotypes (11). A previous study has proposed six dysfunctional brain circuits in MDD, namely default mode, salience, negative affect, positive affect, attention, and cognitive control (11). Figure 2 is an illustrative summary of these dysfunctional brain circuits and their associations with WMT modulated by different DBS targets in MDD. FM and CB are implicated in the default mode circuit; UF is implicated in the salience circuit; CB, UF, and FM are implicated in the negative affect circuit; CB, UF, FM, MFB, and IFOF are implicated in the positive affect circuit; CB, SLF, and IFOF are implicated in the attention circuit; CB and SLF are implicated in the cognitive control circuit. We further present a taxonomy of DBS targets based on their associated WMT and modulated dysfunctional brain circuits: SCG, NAcc, and VC/VS modulate default mode, salience, and negative affect circuits simultaneously; SCG, Nacc, VC/VS, BNST, ITP, and LH modulate attention circuit; All of the targets modulate positive affect circuit, and only SCG target modulates cognitive control circuit. In summary, SCG are implicated in all of the dysfunctional brain circuits modulation, Nacc and VC/VS are implicated in modulating most of the dysfunctional brain circuits except cognitive control circuit, and other targets are mainly implicated in modulation of positive affect and attention circuits. We hypothesize that DTI-based personalized targeting strategy will render more favorable clinical outcome for each subtype of MDD. For example, abnormality is reported in CB, IFOF, and SLF in MDD with cognitive deficit, and SCG, based on DTI studies, may be the optimal target of DBS for this subtype (123). Abnormality in CB, UF, SLF, IFL, IFOF is reported in MDD with attention deficit, and multiple DBS targets are implicated in these networks, including SCG, NAcc, VC/VS, BNST, ITP, and LH (125). However, the majority of DTI studies reported mixed results from different subtypes of MDD, and we are not yet at a stage where we can pinpoint a DBS target based on individual clinical manifestations. Further DTI studies underlying different subtypes of MDD is warrant for optimizing DBS targets.

## Conclusions and Perspectives

In this review, we focus on seven currently used DBS targets for MDD. Most of these clinical trials show that DBS has potential efficacy in the treatment of MDD, though the outcomes are inconsistent. Well-documented and well-designed double-blind RCTs are necessary to provide more powerful evidence of the efficacy and safety of DBS for MDD. Trials comparing various combinations of stimulation parameters and symptoms improvement to identify optimal stimulation parameters are also required (131). In addition, lack of personalized targeting is another important factor that should be taken into account for suboptimal outcomes. A recent study reported the use of intracranial electrophysiology and focal electrical stimulation to identify personalized treatment location (9). In this review, we propose a taxonomy of DBS targets for MDD that may help clinicians to choose the personalized DBS target by non-invasive methods, such as functional magnetic resonance imaging, diffusion tensor imaging, and positron-emission tomography. Among these techniques, DTI is important for understanding the network mechanism and development of MDD, which reveals widespread structural connectivity dysfunctions in MDD. Recently, a study of WMT abnormalities in different subgroups of MDD patients suggested a novel pathway to understanding the heterogeneity of MDD, and may shed light on optimization of subtype-specific treatment approaches (125). Longitudinal study, such as evaluating the time-dependent changes of WMT structures in MDD before and after DBS, may lead to a better understanding of the action mechanisms of DBS. Finally, the identification of individual clinical characteristics and specific WMT abnormalities could serve as a biomarker of therapeutic response in future DBS for MDD studies.

## Author Contributions

QY, XG, and ZZhu performed the literature review and drafted the manuscript. ZZhe, CF, and HJ collected the data and revised the manuscript. JZha, JZhu, and HW supervised all aspects of the study and revised the manuscript. All authors reviewed the manuscript and approved the final version.

## Funding

This work was supported by the National Natural Science Foundation of China (General Program 82171519), Academy of Science and Technology of Zhejiang University Program (K20210252), and ZJU-XITOU Brain-Machine Intelligence Research Center.

## Conflict of Interest

The authors declare that the research was conducted in the absence of any commercial or financial relationships that could be construed as a potential conflict of interest.

## Publisher's Note

All claims expressed in this article are solely those of the authors and do not necessarily represent those of their affiliated organizations, or those of the publisher, the editors and the reviewers. Any product that may be evaluated in this article, or claim that may be made by its manufacturer, is not guaranteed or endorsed by the publisher.
