# Supplementary material for: White Matter Tracts Associated With Deep Brain Stimulation Targets in Major Depressive Disorder: A Systematic Review
Source: Front Psychiatry. 2022 Apr 28;13:806916. doi: 10.3389/fpsyt.2022.806916 (PMC9095936; doi:10.3389/fpsyt.2022.806916)
Supplement: Supplementary file 1 [file Table_1.DOCX]

**Table 1 The targets of DBS for depression**

| Reference | No. of Patients, age | | Follow-up | | | Study type | | Electrode type | | Stimulation parameters | | | | | Responder | | Remission | Nonresponder |
| --- | --- | --- | --- | --- | --- | --- | --- | --- | --- | --- | --- | --- | --- | --- | --- | --- | --- | --- |
|  | **Pulse width Amplitude Frequency**  **(μs) (mA/V) (Hz)** | | | | | | | | | | | | | | | |  |  |
| **SCG** | | | | | | | | | | | | | | | | | | |
| Sankar et al. 2020[23] | 27 (47.2y) | | 12 months | | | | RCT | BL, MP | | 90 | 3-6 V | | 130 | | 55.6% (15/27) | | - | 44.4% (12/27) |
| Crowell et al. 2019[32] | 28 (45y) | | 2-8 years | | | | OLS | BL | | 91/87 | 5-9mA | | 130 | | ≥50% | | ≥30% | - |
| Eitan et al.  2018 [147] | 9(46y) | | 12 months | | | | RCT | BL,MP | | 91 | 4-8mA | | 20or130 | | 44.4%(4/9) | | - | 55.6%(5/9) |
| Merkl et al.  2018[36] | 8(48.3y±12.89) | | 28 months | | | | RCT | BL,MP | | 90 | 2.5-10V | | 130 | | 6months:  37.5%(3/8)  12months:  43%(3/7)  24months:  33.3%(2/6) | | 6months:  12.5%(1/8)  12months:  14.2%(1/7)  28months:  33.3%(2/6) | 6months:  50%(4/8)  12months:  57.1%(4/7)  24months:  66.7%(4/6)  28months:  66.7%(4/6) |
| Riva-Posse et al.  2018[15] | 11(48.7y±10.0) | | 12months | | | | RBT  OLS | BL,MP | | 91 | 6-8V | | 130 | | 81.8% (3/11) | | 54.5% (6/11) | 18.2%(2/11) |
| Holtzheimer et al.  2017[148] | 90(50.5y±9.73) | | 24 months | | | | RCT | BL,MP | | 91 | 4-8mA | | 130 | | 12months:  28.9%(26/90)  18months:  45.6%(41/90)  24months:  42.2%(38/90) | | 12months:  14.4%(13/90)  18months:  15.6%(14/90)  24months:  22.2%(20/90) | 12months:  56.7%(51/90)  18months:  38.9%(35/90)  24months:  35.6%(32/90) |
| McInerney et al.  2017[21] | 20(47.4y±10.4) | | 12months | | | | OLS | BL,MP | | 90 | 2.5- 9V | | 130 | | 55%(11/20) | | - | 45%(9/20) |
| Funayama et al.  2016[24] | 1(69y) | | - | | | | CR | - | | - | - | | - | | √ | | - | - |
| Accolla et al.  2016[38] | 5(45.2y±28.8) | | 24months | | | | OLS | BL,MP | | 90 | 5V | | 130 | | 20%(1/5) | | - | 80%(4/5) |
| Richieri et al.  2016[28] | 1(52y) | | 6months | | | | CR | BL,BP | | 90 | 4.2V | | 130 | | √ | | - | - |
| Puigdemont et al.  2015[22] | 5(46.35y±12.1) | | 6months | | | | DBC  RCT | BL,BP | | 120-240 | 3.5- 5V | | 130-135 | | - | | Active stimulation:  80%(4/5)  Sham stimulation:  40%(2/5) | Active stimulation:  20%(1/5)  Sham stimulation:  60%(3/5) |
| Perez-Caballero et al.2014[149] | 8（-） | | 1month | | | | OLS | BL,MP | | 120-210 | 3.5-5V | | 135 | | Not receive  analgesic-anti-inflammatory drugs:  1week:  50%(2/4)  4week:  25%(1/4)  Receive  analgesic-anti-inflammatory drugs:  1week:  25%(1/4)  4weeks:0 | | Not receive  analgesic-anti-  inflammatory drugs:  1week:  50%(2/4)  4week:  75%(3/4)  Receive analgesic-anti- inflammatory drugs :  1week:  50%(2/4)  4weeks:0 | Not receive  analgesic-anti-inflammatory drugs:  1week:  50%(2/4)  4week:0  Receive analgesic-anti-inflammatory drugs :  1week:  25%(1/4)  4weeks:  100%（4/4） |
| Torres et al.  2013[27] | 1(78y) | | 9months | | | | CR | BL,MP | | 91 | 6mA | | 130 | | √ | | - | - |
| Merkl et al.  2013[34] | 6(50.7y) | | 24-36weeks | | | | OLS | BL,MP | | 90 | 2.5-10V | | 130 | | 66.6%(2/6) | | 33.3%(2/6) | - |
| Ramasubbu et al.   2013[150] | 4(50.25y±7.26) | | 36weeks | | | | OLS | BL,MP | | 60-450 | 0-10.5V | | 2-185 | | 75%(3/4) | | - | 25%(1/4) |
| Holtzheimer et al.  2012[31] | 17(42y±8.9) | | 24months | | | | OLS | BL,MP | | 91 | 4-8V | | 130 | | 24weeks:  41%,(7/17)  1 year:  36%(5/14)  2years :  33.3%(4/12) | | 24weeks:  18%(3/17)  1year:  36%(5/14)  2year:  58%(7/12) | 24weeks:  41.2%(7/17)  1year:  25.6%(4/14)  2year:  8.3%(1/12) |
| Puigdemont et al.   2012[20] | 8(47.4y±11.3) | | 12months | | | | OLS | BL,BP | | 90 | 3.6V | | 135 | | 12.5%(1/8) | | 50%(4/8) | 37.5%(3/8) |
| Broadway et al.   2012[19] | 12(40.4y±9.78) | | 4-24weeks | | | | OLS | BL,MP | | 90 | 6-8V | | 130 | | 50%(6/12) | | - | 50%(6/12) |
| Lozano et al.  2012[33] | 21(47.3y±6.1) | | 12months | | | | OLS | BL,MP | | 91-100.5 | 4.2-5.2mA | | 128.1-130.5 | | 1month:  57%(12/21)  6months:  48%(10/21)  12months:  29%(6/21) | | - | 1month:  43%(9/21)  6months:  52%(11/21)  12months:  71%(15/21) |
| Kennedy et al.  2011[18] | 20(47.4y±10.4) | | 36-72months | | | | OLS | BL,MP | | 90 | 3.5-5V | | 130 | | 1year:62.5%  2 years:46.2%  3 years:75%  Last follow-up  (mean) :64.3%. | | Last follow-up:  >=33.3% | - |
| Holtzheimer,et al.  2010[29] | 1(27y) | | 6months | | | | CR | BL,MP | | 91 | 6mA | | 130 | | √ | | - | - |
| Guinjoan et al.  2010[25] | 1(60y) | | 18months | | | | CR | UL ,MP | | 90 | 4.5V | | 120 | | √ | | - | - |
| Puigdemont et al.  2009[26] | 1(64y) | | 12months | | | | CR | BL,MP | | 90 | 3.6V | | 135 | | - | | √ | - |
| Hamani et al.  2009[17] | 20(-) | | 12months | | | | OLS | BL,MP | | 90 | 3-5V | | 130 | | 55%(11/20) | | - | 45%(9/20) |
| Neimat et al.  2008[151] | 1(55y) | | 30months | | | | CR | MP | | 60 | 4.5V | | 130 | | - | | √ | - |
| Lozano et al.  2008[152] | 2(47.4y±10.4) | | 12months | | | | OLS | BL,MP | | 90 | 3.5- 5V | | 130 | | 60%(12/20) | | 35%(7/20) | 5%(1/20) |
| Mayberg et al.  2005[12] | 6(46y±8) | | 6months | | | | OLS | BL,MP | | 60 | 4V | | 130 | | 16.7%(1/6) | | 50%(3/6) | 33.3%(2/6) |
| **MFB** | | | | | | | | | | | | | | | | | | |
| Coenen et al. 2019[53] | 16(51.6y±10.2) | | 12months | | | | RCT | BL,MP | | 60 | 3mA | | 130 | | 50%(8/16) | | 50%(8/16) | - |
| Fenoy et al.  2018[50] | 6(50.2y) | | 52weeks | | | | OLS | BL,BP | | 60 | 3V | | 130 | | - | | 80% (4/5)  (a case of lost visit) | 20%(1/5) |
| Bewernick et al.  2017[51] | 8(41.9y±8.70) | | 12-48months | | | | OLS | BL,BP | | 60 | 2-3V | | 130 | | 25%（2/8） | | 50%（4/8） | 25%(2/8) |
| Blomstedt et al.  2017[52] | 1(60y) | | 24months | | | | CR | BL,BP | | 60 | 2.8-3v | | 130 | | - | | √ | - |
| Fenoy et al.  2016[49] | 4(46.3y±8.9) | | 26weeks | | | | OLS | BL,BP | | 75 | 3-3.5V | | 125 | | 66.7%（2/3） | | - | 33.3%(1/3) |
| Schlaepfer et al.  2013[48] | 7(42.6y±9.8) | | 12-33weeks | | | | OLS | BL,BP | | 60 | 2-3V | | 130 | | 28.6%（2/7） | | 57.1%(4/7) | 14.3%(1/7) |
| **VC/VS** | | | | | | | | | | | | | | | | |  |  |
| Camprodon et al. 2021[72] | 57y | | 32months | | | | CR | BL | | 210 | 7V(left);5V(right) | | 130 | | √ | | - | - |
| Bergfeld et al.  2017[73] | 25(53.1y±8.4) | | 52weeks (OLS)  12weeks (RCT) | | | | OLS-RCT | BL | | 90 | 2.5- 6V | | 130 -180 | | 60% (15/25) | | - | 40%(10/25) |
| Dougherty et al.  2015[71] | 30(47.7y) | | 16weeks (RCT)  12-24months (OLS) | | | | RCT-OLS | BL,MP | | 90-210 | 8V | | - | | 16weeks:  20%(3/15)  vs14.3%(2/14)  12months:  20%(6/30)  24months:  23.3%(7/30) | | 12months:  13% (4/30)  24months:  20%(6/30) | - |
| Strong et al.  2012[70] | 1(43y) | | 48months | | | | CR | MP | | 120 | 6V | | 130 | | √ | | - | - |
| Malone et al.  2010[68] | 17(46.3y) | | 14-67months | | | | OLS | BL,MP | | - | 2.5-8V | | 100 -130 | | 3months:  53%(9/17)  6months:  47%(8/17)  12months:  53%(9/17)  Last follow-up:  35%(6/17) | | 3months:  35%(6/17)  6months:  29%(5/17)  12months:  41%(7/17)  Last follow-up:  35%(6/17) | 3months:  12%(2/17)  6months:  24%(4/17)  12months:  6%(1/17)  Last follow-up:  30%(5/17) |
| Malone et al.  2009[69] | 15(46.3±10.8) | | 12months | | | | OLS | BL,MP | | 113 | 6.7V | | 127 | | 53.3%(8/15) | | 40%(6/15) | - |
| **Nacc** | | | | | | | | | | | | | | | | |  |  |
| Millet et al.  2014[60] | 6(55.5y) | | 4months | | | | OLS | BL,MP | | 60 | | 4-8V | 130 | | - | | - | 100%(6/6) |
| Bewernick et al.  2012[61] | 11(48.3y±11.8) | | 12-48months | | | | OLS | BL,MP | | 90 | | 5-8V | 130 | | 45.5%(5/11) | | - | 54.5%(6/11) |
| Bewernick et al.  2010[62] | 10(48.6y±11.7) | | 12months | | | | OLS | BL,MP | | 60-210 | | 1.5-10V | 100-150 | | 20%(2/10) | | 30%(3/10) | 50%(5/10) |
| **BNST** | | | | | | | | | | | | | | | | | | |
| Fitzgerald et al.  2018[82] | | 5(44.6y±12.3 ) | | 18-24months | | | OLS | | BL | 120 | | 1-7V | 130 | | 12months:  20%（1/5） | 6months:  20%（1/5）  12months:  20%（1/5）  Last follow-up:  60%（3/5） | | 6months:  80%（4/5）  12months:  60%（3/5）  Last follow-up:  40%（2/5） |
| Cassimjee et al.  2018[81] | | 1(36y) | | 12months | | | CR | | BL,BP | 240 | | 3.4V | 130 | | - | √ | | - |
| Blomstedt et al.  2017[52] | | 1(60y) | | 12months | | | CR | | BL,BP | 120 | | 4.3V | 130 | | - | √ | | - |
| **ITP** | | | | | | | | | | | | | | | | | | |
| Raymaekers et al.  2017[90] | | 7(50.0y±5.6) | | 92months | | | DBC | | BL | - | | - | - | 43% (3/7) | | 14%(1/7) | | 43% (3/7) |
| Jiménez et al.  2013[88] | | 1(-) | | 3years | | | CR | | BP | 450 | | 3-5V | 130 | √ | | - | | - |
| Jiménez et al.  2007[89] | | 1(-) | | 18months | | | CR | | BP | 450 | | 3-5V | 130 | √ | | - | | - |
| Jiménez et al.  2005[87] | | 1(49y) | | 24months | | | CR | | BP | 450 | | 2.5V | 130 | √ | | - | | - |
| **LH** | | | | | | | | | | | | | | | | | | |
| Wang et al. 2020 [97] | | 1(34y) | | 12 weeks | CR | | | | BL | 90 | | 0-10V | 160 | √ | | | - | - |
| Sartorius et al.  2010[95] | | 1(64y) | | 15months | CR | | | | BL | - | | 10.5V | - | - | | | √ | - |

Abbreviations: BL, bilateral; BNST, bed nucleus of the stria terminalis; BP, bipolar; CR, case report; DBS, deep brain stimulation; DBC, double blind crossover; ITP, inferior thalamic peduncle; LH, lateral habenula; MFB, medial forebrain bundle; MP, monopolar; NAcc, nucleus accumbens; OLS, open-label study; QP, quadripolar; RBT, randomized blind trial; RCT, randomized controlled trial; SCG, subcallosal cingulate gyrus; TRD, treatment-resistant depression; UL, unilateral; VC/VS, ventral capsule-ventral striatum; ‘-’data not provided; ‘√’data provided with results.
