# Supplementary material for: White Matter Tracts Associated With Deep Brain Stimulation Targets in Major Depressive Disorder: A Systematic Review
Source: Front Psychiatry. 2022 Apr 28;13:806916. doi: 10.3389/fpsyt.2022.806916 (PMC9095936; doi:10.3389/fpsyt.2022.806916)
Supplement: Supplementary file 2 [file Table_2.DOCX]

Supplementary Table 2 DTI Studies in Depression

| Reference | Participant types | Participants | Results |
| --- | --- | --- | --- |
| Wang et al. 2020^1^ | Late-onset depression  (cognitive deficit) | 38LOD(70.0)  32HC(70.0) | Decreased FA in the left posterior CB, right IFOF and SLF.  Reduced nodal efficiencies in the frontal-striatal-occipital and posterior default-mode regions. |
| Liang et al. 2019^2^ | MDD  (group1: deficit in attention and memory  group2: delayed memory  group3: no significant deficit) | 116MDD(26.1)  118HC(26.0) | Gourp1: decreased FA in CB, CC, bilateral SLF, bilateral internal capsule, bilateral posterior thalamic radiation, CST, bilateral UF, ILF and IFOF. Widespread disruption.  Group2: part of the genu, body, splenium of the CC, and left ILF  Group3: no significant abnormal |
| Zheng et al. 2019^3^ | MDD | 27MDD(35.7±11.0)  31HC(32.2±11.4) | Decreased FA in ILF and CB.  Deficit in cortical-limbic network, frontal-cingulate network, default mode network, cognitive network, and affective network. |
| Bhatia et al, 2018^4^ | FED and TRD | 103MDD (34.5±18.1) 74HC (39.2±16.7) | Decreased FA in subgenual and polar stem of UF, as well as the subgenual and amygdaloid CB.  No significant difference in first-episode and chronic depression. |
| Sugimoto et al, 2018^5^ | FED | 35MDD (46.3±14.3) 35HC (44.0±11.4) | Decreased FA in the bilateral CST, bilateral, IFOF, left ATR, and genu of the CC (FM). |
| Deng et al, 2018^6^ | MDD | 36MDD (29.5±8.6) 45HC (28.3±9.5) | Decreased FA in left ATR, left CST, and left FM  Increased FA in left SLF, and left ILF |
| Tymofiyeva et al, 2017^7^ | Adolescent depression | 57AD (16.2±1.3) 41HC (16±1.4) | Decreased FA in connections between right caudate and frontal gyri (superior, middle, and inferior), insula, and anterior cingulate  Connectivity between right caudate and MFG was negatively correlated with total stress. |
| Vilgis et al, 2017^8^ | Adolescent depression | 25 HRD (12.1 ± 1.8) 25 HC (11.9 ± 1.0) | Decreased FA in left CST, left IFOF and left UF  Left UF is negative association with self-reported symptoms of depression. |
| Yang et al, 2017^9^ | FED | 30 MDD (29.3±8.4) 28 HC (28.6±6.9) | Decreased FA in left CB and FM  Increased RD in bilateral ATR, CST, SLF, and left UF  Anhedonia is correalated with reduced RD. |
| Repple et al, 2017^10^ | Unipolar depression | 43 UD (34.2±12.0) 42 HC (36.1±11.3) | Decreased FA in right CST and right IC |
| Srivastava, 2016^11^ | FED | 15 MDD (30.1)  15 HC (29.1) | Decreased FA in left SLF, right lentiform, right hippocampus, and left parietal region. |
| Chen et al, 2016^12^ | MDD | 16 MDD (44.8 ± 2.2)  47 HC (45.0 ± 1.88) | Decreased FA in SLF.  Disruptions in connectivity in the bilateral frontal lobes.  Depression score (HADS) is negtively correlated with CC. |
| Liu et al, 2016^13^ | FED | 29 MDD (45.7 ± 12.5)  47 HC (41.8 ± 11.0) | Decreased FA in the bilateral CST, left IFOF, left UF, left ATR, left EC and right SCR |
| Yamada et al, 2015^14^ | MDD  (cognitive performance) | 18 MDD(45.7±7.7) 21 HC(41.2±10.0) | Decreased FA in anterior CC  The FA of CC is significantly corelated with the digits equencing task and symbol coding in the MDD group. |
| Jiang et al, 2015^15^ | FED | 35 MDD(29.5±8.6) 34 HC(31.9±8.8) | Decreased FA in right CB (hippocampal) and bilateral ATR  (Papez circuit) |
| Jia et al, 2014^16^ | MDD  (suicide attempts) | 63 MDD(35.2±14.5) 46 HC(33.3±11.4) | Decreased FA in left ALIC projecting to the MFG, OFC and thalamus.  Patients with suicied attempts had greater abnormalities in the left orbitofrontal cortex and thalamus. |
| Adelino et al, 2014^17^ | TRD  RRD  FED | 18 TRD (48.5 ± 7.3)  19 FED (44.2 ± 6.9)  15 RRD (47 ± 9.4)  17 HC (43.4 ± 11.4) | Decreased FA in bilateral IFOF, ILF, SLF, FM, body of CC and CB  Reduced FA in vmPFC predict previous episodes and greater severity of symptom. CC and UF are affected within this area. |
| Bracht, 2014^18^ | Melancholic depression  Non-melancholic depression | 12 MD (50.1±13.9)  10 NMD(38.5 ± 12.0)  21 HC (41.4 ± 13.6) | Decreased FA in right VTA-lOFC and VTA-dlPFC connections between melancholic depression and healthy.  Decresed FA of the pathways correlated negatively with depression scale rating scores. |
| Song et al, 2014^19^ | MDD | 95 MDD(33.8±13.1) 34 HC(31.5±12.4) | Decreased FA in solitary tract (brainstem to amygdala) |
| Lewinn et al, 2014^20^ | Adolescent depression | 52 AD (16 ± 0.2) 42 HC (16 ± 0.2) | Decreased FA in bilateral UF (tractography)  Decreased FA in CC, ACR, SCR, IFOF, IC, and EC (TBSS) |
| Kwaasteniet et al, 2013^21^ | MDD | 18 MDD (44.6 ± 10.4) 24 HC (40.2 ± 13.1) | Decreased FA in UF  Decreased FA in UF is negtively correlated with depression severity. |
| Henderson et al, 2013^22^ | Adolescent depression  (anhedonia and irritability) | 17 AD (16.8 ± 2.2) 16 HC (16.4 ± 1.4) | Decreased FA in anterior CB and ACR.  Anhedonia is associated with decreased FA in ALIC and projection fibers to the orbitofrontal cortex.  Irritability is associated with decreased FA in the stratum, ACR, and projection to prefrontal and temporal cortices. |
| Bessette et al, 2013^23^ | Adolescent depression | 31 AD (17.1 ± 2.4) 31 HC (17.0 ± 2.4) | Decreased FA in bilateral ALIC, PLIC, ACR, SCR, CC, IFOF, right ATR, left PTR, EC, ILF, UF |
| seok, et al, 2013^24^ | MDD | 86 MDD (44.7 ± 12.2) 62 HC (42.1 ± 14.5) | Decreased FA in genu of CC, bilateral FM, anterior CB, left posterior CB, right SLF and PTR |
| Lai et al, 2013^25^ | FED | 44 FED (36.9 ± 5.3) 27 HC (38.3 ± 11.8) | Decreased FA in left SLF and right ATR  Decreased FA is negatively correlated with depression severity in SLF and with illness duration in right SLF and ATR. |
| Peng et al. 2013^26^ | Treatment resistant depression  (young TRD) | 30 TRD (26.8 ± 5.2) 25 HC (28.2 ± 4.9) | Decreased FA in left MFG, limbic lobe uncus and right CPL  negative correlations between symptom scores (BDI) and reduced FA |
| Tha et al, 2013^27^ | Treatment resistant depression  (free of antidepressant medication for at least 6 months) | 19 TRD (38.6 ± 13) 19 HC (36.5 ± 12.5) | Decreased FA in bilateral frontal WM and ALIC |
| Keedwell et al, 2012^28^ | High risk of depression of family history  18 FH+ and 15 family history (FH–). | 18 HRD (22.2)  15 HC (22.1) | Decreased FA in bilateral CB |
| Guo et al, 2012^29^ | FED | 22 FED (28.1 ± 9.9) 19 HC (24.4 ± 4.2) | Decreased FA in bialteral IC, genu of CC, ACR and right EC |
| Guo et al, 2012^30^ | Treatment resistant depression | 23 TRD (27.4 ± 7.7) 19 HC (24.4 ± 4.2) | Decreased FA in right ALIC, body of CC, and bilateral EC |
| Murphy et al, 2012^31^ | MDD  (not/receive an antidepressant) | 45 MDD (42 ± 10.9) 45 HC (37 ± 12.8) | Decreased FA in FM, ILF, CB, UF, and right SLF |
| Zhang et al, 2012^32^ | MDD | 21 MDD (47.7 ± 10.15) 21 HC (48.3 ± 14.3) | Decreased FA in right UF deression severity  decreased FA was observed with aging |
| Arnold, 2012^33^ | Remitted major depressive disorder | 17 RMDD (30.4 ± 1.4) 21 HC (26.9 ± 7.8) | Increased connectivity from amygdala to hippocampus, cerebellum and brain stem |
| Walther et al, 2012^34^ | MDD  (21 medicated patients) | 21 MDD (41 ± 13.7) 21 HC (45 ± 13.7) | Decreased FA in ACR and left ATR |
| Carballedo et al., 2012^35^ | Thirteen of the patients were currently drug- free, 13 received SSRIs, and 11 dual acting substances venlafaxine or mirtazapine; BDNF alleles | 37 MDD (40.4 ± 10)  42 HC (36.3 ± 13) | Decreased FA in bilateral UF |
| Huang et al., 2011^36^ | Adolescent depression | 18 HR (15.7 ± 2.3)  13 HC (15.5 ± 3.0) | Decreased FA in left CB, splenium of CC, SLF, UF, and IFOF |
| Zhu et al, 2011^37^ | Adolescent depression  (FED) | 25 AD (20.5 ± 1.86) 25 HC (20.33 ± 1.68) | Decreased FA in left ALIC, right parahippocampal gyrus and left posterior cingulate cortex (PCC). |
| Korgaonkar et al, 2011^38^ | Melancholic depression | 11 MD (43.1 ± 15.3) 39 HC (29.6 ± 12.7) | Decreased FA in bilateral fornix-SM, IC, PTR, EC, left SLF, and splenium of CC |
| Wu et al, 2011^39^ | FED | 23 FED (31.4 ± 8.8) 21 HC (30.4 ± 8.2) | Decreased FA in right SLF within the frontal lobe, right middle frontal white matter and left inferior parietal lobe. |
| Ouyang et al, 2011^40^ | FED | 18 MDD (27.4 ± 6.4) 18 HC (27.0 ± 6.0) | Decreased FA in bilateral MFG, right subgyral frontal and temporal lobes, and left middle frontal and cingulate gyri. |
| Cullen et al, 2010^41^ | Adolescent depression  (Generalized Anxiety Disorder (n=7), Social Phobia (n=3), Panic Disorder (n=1) and Post Traumatic Stress Disorder (PTSD) (n=2).) | 14 AD (16.8 ± 1.3) 14 HC (16.8 ± 1.5) | Decreased FA in bilateral UF, IFOF, left anterior CB, and SLF |
| Blood et al, 2010^42^ | MDD | MDD (36.3 ± 12) HC (35.3 ± 11) | Decreased FA in MFB and dlPFC white matter |
| Abe et al, 2010^43^ | MDD  (unipolar major depressive disorder) | 21 MDD (48 ± 13.5)  42 HC (48 ± 13.2) | No significant difference between the two groups for FA |

**Reference**:

1. Wang Z, Yuan Y, You J, Zhang Z. Disrupted structural brain connectome underlying the cognitive deficits in remitted late-onset depression. *Brain Imaging Behav*. Oct 2020;14(5):1600-1611. doi:10.1007/s11682-019-00091-x

2. Liang S, Wang Q, Kong X, et al. White Matter Abnormalities in Major Depression Biotypes Identified by Diffusion Tensor Imaging. *Neurosci Bull*. Oct 2019;35(5):867-876. doi:10.1007/s12264-019-00381-w

3. Zheng K, Wang H, Li J, et al. Structural networks analysis for depression combined with graph theory and the properties of fiber tracts via diffusion tensor imaging. *Neurosci Lett*. Feb 16 2019;694:34-40. doi:10.1016/j.neulet.2018.11.025

4. Bhatia KD, Henderson LA, Hsu E, Yim M. Reduced integrity of the uncinate fasciculus and cingulum in depression: A stem-by-stem analysis. *Journal of affective disorders*. Aug 1 2018;235:220-228. doi:10.1016/j.jad.2018.04.055

5. Sugimoto K, Kakeda S, Watanabe K, et al. Relationship between white matter integrity and serum inflammatory cytokine levels in drug-naive patients with major depressive disorder: diffusion tensor imaging study using tract-based spatial statistics. *Translational psychiatry*. Aug 1 2018;8(1):141. doi:10.1038/s41398-018-0174-y

6. Deng F, Wang Y, Huang H, et al. Abnormal segments of right uncinate fasciculus and left anterior thalamic radiation in major and bipolar depression. *Progress in neuro-psychopharmacology & biological psychiatry*. Feb 2 2018;81:340-349. doi:10.1016/j.pnpbp.2017.09.006

7. Tymofiyeva O, Connolly CG, Ho TC, et al. DTI-based connectome analysis of adolescents with major depressive disorder reveals hypoconnectivity of the right caudate. *J Affect Disord*. Jan 1 2017;207:18-25. doi:10.1016/j.jad.2016.09.013

8. Vilgis V, Vance A, Cunnington R, Silk TJ. White matter microstructure in boys with persistent depressive disorder. *Journal of affective disorders*. Oct 15 2017;221:11-16. doi:10.1016/j.jad.2017.06.020

9. Yang XH, Wang Y, Wang DF, et al. White matter microstructural abnormalities and their association with anticipatory anhedonia in depression. *Psychiatry research Neuroimaging*. Jun 30 2017;264:29-34. doi:10.1016/j.pscychresns.2017.04.005

10. Repple J, Meinert S, Grotegerd D, et al. A voxel-based diffusion tensor imaging study in unipolar and bipolar depression. *Bipolar disorders*. Feb 2017;19(1):23-31. doi:10.1111/bdi.12465

11. Srivastava S, Bhatia MS, Bhargava SK, Kumari R, Chandra S. A Diffusion Tensor Imaging Study Using a Voxel-Based Analysis, Region-of-Interest Method to Analyze White Matter Abnormalities in First-Episode, Treatment-Naive Major Depressive Disorder. *The Journal of neuropsychiatry and clinical neurosciences*. Spring 2016;28(2):131-7. doi:10.1176/appi.neuropsych.15050120

12. Chen VC, Shen CY, Liang SH, et al. Assessment of abnormal brain structures and networks in major depressive disorder using morphometric and connectome analyses. *Journal of affective disorders*. Nov 15 2016;205:103-111. doi:10.1016/j.jad.2016.06.066

13. Liu X, Watanabe K, Kakeda S, et al. Relationship between white matter integrity and serum cortisol levels in drug-naive patients with major depressive disorder: diffusion tensor imaging study using tract-based spatial statistics. *The British journal of psychiatry : the journal of mental science*. Jun 2016;208(6):585-90. doi:10.1192/bjp.bp.114.155689

14. Yamada S, Takahashi S, Ukai S, et al. Microstructural abnormalities in anterior callosal fibers and their relationship with cognitive function in major depressive disorder and bipolar disorder: a tract-specific analysis study. *Journal of affective disorders*. Mar 15 2015;174:542-8. doi:10.1016/j.jad.2014.12.022

15. Jiang W, Gong G, Wu F, et al. The papez circuit in first-episode, treatment-naive adults with major depressive disorder: combined atlas-based tract-specific quantification analysis and voxel-based analysis. *PloS one*. 2015;10(5):e0126673. doi:10.1371/journal.pone.0126673

16. Jia Z, Wang Y, Huang X, et al. Impaired frontothalamic circuitry in suicidal patients with depression revealed by diffusion tensor imaging at 3.0 T. *Journal of psychiatry & neuroscience : JPN*. May 2014;39(3):170-7. doi:10.1503/jpn.130023

17. de Diego-Adelino J, Pires P, Gomez-Anson B, et al. Microstructural white-matter abnormalities associated with treatment resistance, severity and duration of illness in major depression. *Psychological medicine*. Apr 2014;44(6):1171-82. doi:10.1017/s003329171300158x

18. Bracht T, Horn H, Strik W, et al. White matter microstructure alterations of the medial forebrain bundle in melancholic depression. *Journal of affective disorders*. Feb 2014;155:186-93. doi:10.1016/j.jad.2013.10.048

19. Song YJ, Korgaonkar MS, Armstrong LV, Eagles S, Williams LM, Grieve SM. Tractography of the brainstem in major depressive disorder using diffusion tensor imaging. *PloS one*. 2014;9(1):e84825. doi:10.1371/journal.pone.0084825

20. LeWinn KZ, Connolly CG, Wu J, et al. White matter correlates of adolescent depression: structural evidence for frontolimbic disconnectivity. *J Am Acad Child Adolesc Psychiatry*. Aug 2014;53(8):899-909, 909 e1-7. doi:10.1016/j.jaac.2014.04.021

21. de Kwaasteniet B, Ruhe E, Caan M, et al. Relation between structural and functional connectivity in major depressive disorder. *Biol Psychiatry*. Jul 1 2013;74(1):40-7. doi:10.1016/j.biopsych.2012.12.024

22. Henderson SE, Johnson AR, Vallejo AI, Katz L, Wong E, Gabbay V. A preliminary study of white matter in adolescent depression: relationships with illness severity, anhedonia, and irritability. *Frontiers in psychiatry*. 2013;4:152. doi:10.3389/fpsyt.2013.00152

23. Bessette KL, Nave AM, Caprihan A, Stevens MC. White matter abnormalities in adolescents with major depressive disorder. *Brain imaging and behavior*. Dec 2014;8(4):531-41. doi:10.1007/s11682-013-9274-8

24. Seok JH, Choi S, Lim HK, Lee SH, Kim I, Ham BJ. Effect of the COMT val158met polymorphism on white matter connectivity in patients with major depressive disorder. *Neuroscience letters*. Jun 17 2013;545:35-9. doi:10.1016/j.neulet.2013.04.012

25. Lai CH, Wu YT. Alterations in white matter micro-integrity of the superior longitudinal fasciculus and anterior thalamic radiation of young adult patients with depression. *Psychological medicine*. Oct 2014;44(13):2825-32. doi:10.1017/s0033291714000440

26. Peng HJ, Zheng HR, Ning YP, et al. Abnormalities of cortical-limbic-cerebellar white matter networks may contribute to treatment-resistant depression: a diffusion tensor imaging study. *BMC psychiatry*. Mar 2 2013;13:72. doi:10.1186/1471-244x-13-72

27. Tha KK, Terae S, Nakagawa S, et al. Impaired integrity of the brain parenchyma in non-geriatric patients with major depressive disorder revealed by diffusion tensor imaging. *Psychiatry research*. Jun 30 2013;212(3):208-15. doi:10.1016/j.pscychresns.2012.07.004

28. Keedwell PA, Chapman R, Christiansen K, Richardson H, Evans J, Jones DK. Cingulum white matter in young women at risk of depression: the effect of family history and anhedonia. *Biological psychiatry*. Aug 15 2012;72(4):296-302. doi:10.1016/j.biopsych.2012.01.022

29. Guo WB, Liu F, Xue ZM, et al. Altered white matter integrity in young adults with first-episode, treatment-naive, and treatment-responsive depression. *Neuroscience letters*. Aug 1 2012;522(2):139-44. doi:10.1016/j.neulet.2012.06.027

30. Guo WB, Liu F, Chen JD, et al. Altered white matter integrity of forebrain in treatment-resistant depression: a diffusion tensor imaging study with tract-based spatial statistics. *Progress in neuro-psychopharmacology & biological psychiatry*. Aug 7 2012;38(2):201-6. doi:10.1016/j.pnpbp.2012.03.012

31. Murphy ML, Carballedo A, Fagan AJ, et al. Neurotrophic tyrosine kinase polymorphism impacts white matter connections in patients with major depressive disorder. *Biological psychiatry*. Oct 15 2012;72(8):663-70. doi:10.1016/j.biopsych.2012.04.015

32. Zhang A, Leow A, Ajilore O, et al. Quantitative tract-specific measures of uncinate and cingulum in major depression using diffusion tensor imaging. *Neuropsychopharmacology : official publication of the American College of Neuropsychopharmacology*. Mar 2012;37(4):959-67. doi:10.1038/npp.2011.279

33. Arnold JF, Zwiers MP, Fitzgerald DA, et al. Fronto-limbic microstructure and structural connectivity in remission from major depression. *Psychiatry Res*. Oct 30 2012;204(1):40-8. doi:10.1016/j.pscychresns.2012.07.010

34. Walther S, Hugli S, Hofle O, et al. Frontal white matter integrity is related to psychomotor retardation in major depression. *Neurobiology of disease*. Jul 2012;47(1):13-9. doi:10.1016/j.nbd.2012.03.019

35. Carballedo A, Amico F, Ugwu I, et al. Reduced fractional anisotropy in the uncinate fasciculus in patients with major depression carrying the met-allele of the Val66Met brain-derived neurotrophic factor genotype. *American journal of medical genetics Part B, Neuropsychiatric genetics : the official publication of the International Society of Psychiatric Genetics*. Jul 2012;159b(5):537-48. doi:10.1002/ajmg.b.32060

36. Huang H, Fan X, Williamson DE, Rao U. White matter changes in healthy adolescents at familial risk for unipolar depression: a diffusion tensor imaging study. *Neuropsychopharmacology : official publication of the American College of Neuropsychopharmacology*. Feb 2011;36(3):684-91. doi:10.1038/npp.2010.199

37. Zhu X, Wang X, Xiao J, Zhong M, Liao J, Yao S. Altered white matter integrity in first-episode, treatment-naive young adults with major depressive disorder: a tract-based spatial statistics study. *Brain research*. Jan 19 2011;1369:223-9. doi:10.1016/j.brainres.2010.10.104

38. Korgaonkar MS, Grieve SM, Koslow SH, Gabrieli JD, Gordon E, Williams LM. Loss of white matter integrity in major depressive disorder: evidence using tract-based spatial statistical analysis of diffusion tensor imaging. *Hum Brain Mapp*. Dec 2011;32(12):2161-71. doi:10.1002/hbm.21178

39. Wu F, Tang Y, Xu K, et al. Whiter matter abnormalities in medication-naive subjects with a single short-duration episode of major depressive disorder. *Psychiatry Res*. Jan 30 2011;191(1):80-3. doi:10.1016/j.pscychresns.2010.09.002

40. Ouyang X, Tao HJ, Liu HH, et al. White matter integrity deficit in treatment-naive adult patients with major depressive disorder. *East Asian Arch Psychiatry*. Mar 2011;21(1):5-9.

41. Cullen KR, Klimes-Dougan B, Muetzel R, et al. Altered white matter microstructure in adolescents with major depression: a preliminary study. *J Am Acad Child Adolesc Psychiatry*. Feb 2010;49(2):173-83 e1. doi:10.1097/00004583-201002000-00011

42. Blood AJ, Iosifescu DV, Makris N, et al. Microstructural abnormalities in subcortical reward circuitry of subjects with major depressive disorder. *PLoS One*. Nov 29 2010;5(11):e13945. doi:10.1371/journal.pone.0013945

43. Abe O, Yamasue H, Kasai K, et al. Voxel-based analyses of gray/white matter volume and diffusion tensor data in major depression. *Psychiatry research*. Jan 30 2010;181(1):64-70. doi:10.1016/j.pscychresns.2009.07.007
