# Supplementary figures and images for: White Matter Tracts Associated With Deep Brain Stimulation Targets in Major Depressive Disorder: A Systematic Review
Source: Front Psychiatry. 2022 Apr 28;13:806916. doi: 10.3389/fpsyt.2022.806916 (PMC9095936; doi:10.3389/fpsyt.2022.806916)

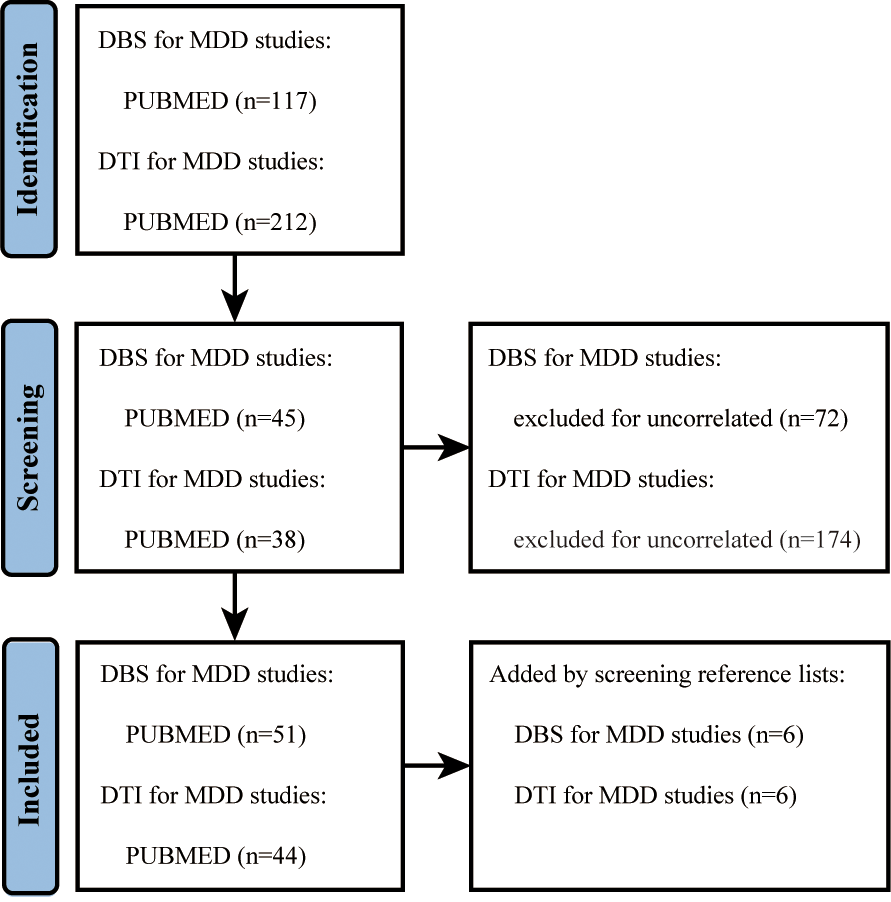

Supplement: Supplementary file 3 [file Image_1.TIF]
